# Metabolic Fate Is Defined by Amino Acid Nature in Gilthead Seabream Fed Different Diet Formulations

**DOI:** 10.3390/ani12131713

**Published:** 2022-07-02

**Authors:** Rita Teodósio, Claúdia Aragão, Luís E. C. Conceição, Jorge Dias, Sofia Engrola

**Affiliations:** 1 Centro de Ciências do Mar (CCMAR), Campus de Gambelas, Universidade do Algarve, 8005-139 Faro, Portugal; rteodosio@ualg.pt (R.T.); caragao@ualg.pt (C.A.); 2 Campus de Gambelas, Universidade do Algarve, 8005-139 Faro, Portugal; 3 SPAROS Lda., Área Empresarial de Marim, Lote C, 8700-221 Olhão, Portugal; luisconceicao@sparos.pt (L.E.C.C.); jorgedias@sparos.pt (J.D.)

**Keywords:** ketogenic, glucogenic, catabolism, amino acid bioavailability, nutrient flux

## Abstract

**Simple Summary:**

The maximisation of fish growth depends on amino acids’ availability in tissues at an optimum ratio since imbalances may lead to their utilisation for energy rather than growth. Amino acids may be catabolised in multiple pathways and be classified according to their metabolic fate: ketogenic and glucogenic. Ketogenic amino acids (e.g., lysine) are precursors of ketone bodies or long chain fatty acids and can be used in lipogenesis. Glucogenic amino acids (e.g., methionine) can be converted into glucose through gluconeogenesis. Some amino acids, such as tryptophan, can be ketogenic and glucogenic. This study aimed to evaluate how fish discriminate among different amino acids when they are fed different diets to attain the best utilisation of the feed. This trial was carried out in gilthead seabream juveniles fed experimental diets containing different levels of protein and/or distinct lipid levels. The metabolic fate and bioavailability of the indispensable amino acids lysine, methionine, and tryptophan were defined by their ketogenic and/or glucogenic nature rather than diet formulations. The optimisation of diets that consider the amino acids’ bioavailability will maximise protein retention in fish and is a viable solution to develop cost-effective fish diets.

**Abstract:**

The sustainability of the Aquaculture industry relies on optimising diets to promote nitrogen retention and maximise fish growth. The aim of this study was to assess how different dietary formulations influence the bioavailability and metabolic fate of distinct amino acids in gilthead seabream juveniles. Amino acids (lysine, tryptophan, and methionine) were selected based on their ketogenic and/or glucogenic nature. Seabream were fed practical diets with different protein (44 and 40%) and lipid contents (21 and 18%): 44P21L, 44P18L, 40P21L, and 40P18L. After three weeks of feeding, the fish were tube-fed the correspondent diet labelled with ^14^C-lysine, ^14^C-tryptophan, or ^14^C-methionine. The amino acid utilisation was determined based on the evacuation, retention in gut, liver, and muscle, and the catabolism of the tracer. The metabolic fate of amino acids was mainly determined by their nature. Tryptophan was significantly more evacuated than lysine or methionine, indicating a lower availability for metabolic purposes. Methionine was more retained in muscle, indicating its higher availability. Lysine was mainly catabolised, suggesting that catabolism is preferentially ketogenic, even when this amino acid is deficient in diets. This study underpins the importance of optimising diets considering the amino acids’ bioavailability and metabolic fate to maximise protein retention in fish.

## 1. Introduction

The sustainability of the aquaculture sector is largely dependent on optimised diets that promote nitrogen retention and maximise fish growth performance. Growth is essentially protein deposition and improved growth requires the knowledge of the ideal dietary amino acid profile [1]. This profile may vary across fish species and developmental stages [2,3,4]. Optimal growth is conditioned by the efficiency of the absorption of each amino acid and the precise knowledge of the rates of absorption and catabolism, i.e., the relative bioavailability of the individual amino acids [5]. The absorption of individual amino acids in the gut relies on a multitude of transport systems, some of which reveal a broad substrate sensitivity; several amino acids may be uptake by the same transporter and one amino acid may be transported by more than one system. Therefore, the absorption of amino acids is influenced by several factors, such as their concentration in the intestinal lumen, transport affinity and capacity for each transporter, and the amount of each transporter present in the epithelium [4,6]. To maximise fish growth, amino acids must be available in tissues at an optimum ratio, as imbalances will lead to amino acid losses for energy purposes rather than for protein synthesis [7,8].

Amino acids may be catabolised in multiple pathways and are classified into two categories according to their metabolic fate: ketogenic or glucogenic, while some amino acids are both ketogenic and glucogenic. Ketogenic amino acids, such as lysine, are catabolised only to produce acetyl-CoA, a precursor of ketone bodies or long-chain fatty acids. Lysine is mainly involved in protein synthesis. However, this indispensable amino acid also plays a role in the structure and function of collagen through its metabolite hydroxylysine, and along with methionine, is required for the synthesis of carnitine. Glucogenic amino acids (e.g., methionine) can generate metabolic intermediate molecules such as alpha-keto acids, pyruvate, and oxaloacetate that are crucial to many processes that occur in animals, and can be converted into glucose through gluconeogenesis [9]. Methionine is a sulphur-containing amino acid involved in protein synthesis, transmethylation, remethylation, and transsulfuration reactions. Additionally, ketogenic plus glucogenic amino acids, such as tryptophan, can be catabolised into both acetyl-CoA and 4–5 carbon unit metabolites. Tryptophan is the least abundant amino acid in fish proteins. As a precursor of the neurotransmitter serotonin and of the hormone melatonin, it regulates stress and immune, as well as behavioural responses in fish [10,11].

Fish feeds contain a fair amount of vegetable ingredients as protein sources. Feeding fish with diets that incorporate considerable amounts of terrestrial plant ingredients has proven its feasibility, even when feeding carnivorous fish species such as the gilthead seabream (*Sparus aurata*), as long as crystalline amino acids are added to overcome any deficiency or imbalance in the amino acid profile [7,12,13,14]. If imbalances occur, amino acid catabolism and consequent metabolic losses are inevitable and excessive nitrogen may be lost to the aquatic environment [15,16,17]. Hence, it is important to understand how fish discriminate among the distinct amino acids when fed different dietary formulations to ensure an optimised nitrogen utilisation under any diet, rearing condition, and developmental stage. The gathered knowledge will help to formulate balanced diets that will allow fish to reach their full growth potential.

The aim of the present study was to assess how different dietary formulations could influence the bioavailability and metabolic fate of selected indispensable amino acids including lysine, tryptophan, and methionine. Indispensable amino acids were selected based on their ketogenic and/or glucogenic nature. Gilthead seabream juveniles were fed practical diets incorporating different protein and/or lipid levels. Metabolic flux assays were performed via tube-feeding ^14^C-labelled diets to estimate evacuation, retention, and catabolism of amino acids.

## 2. Materials and Methods

### 2.1. Experimental Diets

Four experimental diets were formulated with two levels of crude protein (44 and 40% CP) and two levels of crude lipids (21 and 18% CL) using practical ingredients. Protein and lipid contents were based on the range used in commercial diets for gilthead seabream juveniles. Diets were designated 44P21L, 44P18L, 40P21L, and 40P18L according to their protein and lipid contents (Table 1). High protein diets, 44P21L and 44P18L, included 27% fishmeal and incorporated 51–54% plant ingredients as protein sources. The lower protein diets (40P21L and 40P18L) contained 21% fishmeal and 57–60% plant protein sources. Fish oil to rapeseed oil ratio was kept at approximately 1.5 to 1.0 in all diets. Higher protein diets (44P21L and 44P18L) were formulated to meet lysine requirements for gilthead seabream juveniles, while lower protein diets (40P21L and 40P18L) were formulated to be deficient in lysine. Formulation, proximate composition, and amino acid analysis of diets are presented in Table 1 and Table 2.

All experimental diets were manufactured by SPAROS Lda. (Olhão, Portugal). Diets (pellet size 2 mm) were produced via extrusion by means of a pilot-scale twin-screw extruder (CLEXTRAL BC45; Clextral, Firminy, France) with a screw diameter of 55.5 mm and temperature ranging from 105 °C to 110 °C. Upon extrusion, all batches of extruded feeds were dried in a vibrating fluid bed dryer (model DR100; TGC Extrusion, Roullet-Saint-Estèphe, France) and cooled at room temperature. Subsequently, the oil fraction was added under vacuum coating in a Pegasus vacuum mixer (PG-10VCLAB; DINNISSEN, Sevenum, The Netherlands). Experimental diets were kept in a cool and aerated storage room.

### 2.2. Fish Husbandry

Throughout the conditioning feeding period, gilthead seabream (*Sparus aurata*) juveniles were maintained at the Centre of Marine Sciences (CCMAR, Faro, Portugal). Fish were reared in 40 L cylinder-conical tanks in a recirculation aquaculture system at an initial density of 4.8 kg m^−3^ (45 fish per tank). Water temperature and salinity were 20.1 ± 1.2 °C and 32.2 ± 1.4 ppt, respectively. Fish were assigned one of the four experimental diets and fed by automatic feeders, six times per day at a feeding ration of 3% body weight day^−1^, for three weeks (six meals of 0.5% body weight per day).

### 2.3. Metabolic Flux Assays

The metabolic fate of the indispensable amino acids as a function of their nature (ketogenic and/or glucogenic) and of the dietary treatment was determined after three weeks of feeding the experimental diets. The metabolic flux assays were performed according to the methodology previously published in detail by Teodósio et al. [18]. This method was adapted from Costas [19], which was modified for juvenile fish from the procedure described by Rønnestad et al. [20], a modification from the original method published by Rust et al. [21].

Briefly, after being fasted for 24 h, anaesthetised fish (50 mg L^−1^ 2-phenoxyethanol, Merck KGaA, Darmstadt, Germany) from each dietary treatment (44P21L, 44P18L, 40P21L, and 40P18L) were tube-fed with the correspondent experimental diet labelled with one of the following tracers: ^14^C-lysine ([U-^14^C]-L-lysine; 1.85 MBq, Perkin Elmer, Waltham, MA, USA), ^14^C-tryptophan ([1-^14^C]-L-tryptophan; 1.85 MBq, American Radiolabeled Chemicals Inc., St. Louis, MO, USA) or ^14^C-methionine ([1-^14^C]-L-methionine; 1.85 MBq, American Radiolabeled Chemicals Inc.). Fish were tube-fed the number of pellets corresponding to a single meal (approximately 0.5% of fish body weight). The tube-fed tracer presented no nutritional value and its metabolic fate was considered to represent the fate of the tracee of the diets [22]. Six fish per diet and tracer (average weight of 9.1 ± 2.9 g; mean ± standard deviation) were subjected to this procedure (6 fish × 4 diets × 3 tracers, *n* = 72 fish in total).

Tube-fed fish were transferred into individual incubation chambers containing 2 L of seawater at 20 °C. Chambers were hermitically sealed and each individual chamber was connected to a series of ^14^CO_2_-metabolic traps to collect the ^14^CO_2_ produced by the fish from catabolism of ^14^C-amino acids. After 18 h, fish were euthanised inside the metabolic chamber by a lethal dose of anaesthetic (1000 mg L^−1^ of MS-222 buffered with sodium bicarbonate) and removed for tissue sampling. After fish were removed, the chambers were resealed, and the incubation seawater was acidified gradually leading to any remaining ^14^CO_2_ in the seawater to be trapped in the ^14^CO_2_-metabolic traps. The acidification procedure published by Rønnestad et al. [20] allowed for discrimination between unabsorbed nutrients evacuated from the gut and molecules originating from catabolism of the absorbed nutrient, both of which were present in the incubation water.

#### Metabolic Budget Determination

After acidification of the incubation water, seawater samples from each chamber (*n* = 5) and individual samples from each ^14^CO_2_-metabolic trap (*n* = 3) were collected for radioactivity (disintegrations per minute, DPM) determination. The amount of radioactivity present in the seawater resulted from evacuated (unabsorbed) ^14^C-amino acids or from a negligible amount of other metabolites containing the ^14^C-skeleton, while the radioactivity present in the ^14^CO_2_-metabolic traps resulted from ^14^C-amino acid catabolism.

From each fish, the gastrointestinal tract (gut, from the oesophagus to the hindgut), whole liver, and whole left side skin-on fillet (muscle) were sampled to assess ^14^C-amino acid retention in the body fractions. Tissues sampled in this trial represented between 40–45% of the total weight of fish. Fish digestive tract was previously washed in Ringer solution for marine fish to ensure that no alimentary bolus was present before DPM determination. All fish tissues were fully dissolved by adding an appropriate volume of Solvable™ (Perkin Elmer) at 50 °C for 24 h. Gut and liver were analysed as whole whereas two samples of dorsal muscle were used for DPM counting. Ultima Gold XR scintillation cocktail (Perkin Elmer) was added to all samples (incubation seawater, ^14^CO_2_-metabolic traps, gut, liver, and muscle fractions) and DPM determined in a TriCarb 2910TR low activity liquid scintillation analyser (Perkin Elmer). Samples were corrected for quench and lumex.

Amino acid utilisation in seabream juveniles as a function of the amino acids’ nature and diet formulation was determined based on the percentage of ^14^C-amino acid evacuated, retained in the different tissues, or catabolised, as follows:

$$Evacuation\;\left(\%\right)=\;(DPM_{SW}/DPM_{Total})\times 100\;Gut\;Retention\;\left(\%\right)=\;(DPM_{Gut}/DPM_{Total})\times 100\;Liver\;Retention\;\left(\%\right)=\;(DPM_{Liver}/DPM_{Total})\times 100\;Muscle\;Retention\;\left(\%\right)=\;(DPM_{Muscle}/DPM_{Total})\times 100\;Catabolism\;\left(\%\right)=\;(DPM_{Traps}/DPM_{Total})\times 100$$

where *DPM_Total_* is the sum of the radioactivity (DPM) determined in the incubation seawater (*DPM_SW_*), gut (*DPM_Gut_*), liver (*DPM_Liver_*), muscle (*DPM_Muscle_*), and ^14^CO_2_-metabolic traps (*DPM_Traps_*) fractions.

### 2.4. Chemical Analysis

Experimental diets were finely ground and analysed in duplicate for dry matter, ash, crude protein (N × 6.25), crude lipid, gross energy, and phosphorus contents, following standard procedures of the Association of Official Analytical Chemists [23]. Diets’ total amino acid profile was determined by ultra-high-performance liquid chromatography (UPLC), after acid hydrolysis. Tryptophan content was not determined since it is partially destroyed by acid hydrolysis. All analyses were performed as described by Teodósio et al. [24].

### 2.5. Data Analysis

Data from the metabolic flux assays are presented as mean ± standard deviation. Data expressed as a percentage were arcsine square root transformed prior to the statistical analysis [25]. All data were checked for normal distribution and homogeneity of variances. The experimental design was randomised in a 4 × 3 factorial design with four dietary treatments (44P21L, 44P18L, 40P21L, and 40P18L) and three dietary components (lysine, tryptophan, and methionine). Main and interaction effects were identified by two-way analysis of variance (two-way ANOVA) followed by Tukey’s multiple-comparison test at *p* < 0.05 level of significance. Statistical analyses were performed using the IBM SPSS Statistics 26 software (IBM Corp, Armonk, NY, USA).

Additionally, a principal component analysis (PCA) was performed to confirm differences between diet formulations and amino acids’ nature and find potential clusters of observations. The standard prcomp R function in the auto-scaled matrices was used for PCA and score plots were produced for the two first principal components (PC1 and PC2) using the ggbiplot package for R. Loadings for PC1 and PC2 were calculated to determine the weight of each original variable in the corresponding PCs. All analyses were carried out using the open-source software R version 4.0.4 (R Core team).

To assess any dietary amino acid imbalances of the experimental diets, dietary A/E ratios [26] were calculated, on a weight basis, as: [each indispensable amino acid (IAA) content × (total IAA content)^−1^ × 1000], and plotted against previously published A/E ratios for gilthead seabream juveniles [27]. Cysteine and tyrosine were included with the IAA, since they can only be synthesised from methionine and phenylalanine, respectively. A deficiency or excess for a given IAA calculated as: [(A/E_Diet_ − A/E_Fish_) × A/E_Fish_^−1^] × 100, was assumed to occur when the dietary A/E ratio was at least 10% lower or higher than that of the fish.

## 3. Results

**Figure 1:**
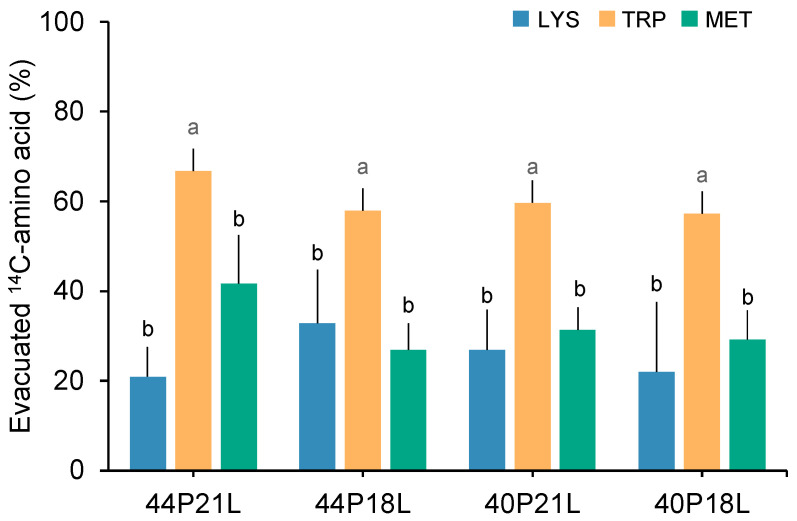
Proportion (%) of the total recovered ^14^C-amino acids lysine (LYS), tryptophan (TRP) and methionine (MET) that was evacuated in gilthead seabream juveniles fed 44P21L, 44P18L, 40P21L, or 40P18L diets. Values are presented as means ± standard deviation (*n* = 6 fish for each diet and tracer). Letters (a and b) represent significant differences among amino acids’ metabolic fate as a function of their nature (two-way ANOVA followed by Tukey’s multiple-comparison test, *p* < 0.05).

In all the dietary treatments, the proportion of the tube-fed ^14^C-tryptophan that was evacuated was significantly higher compared to ^14^C-lysine or ^14^C-methionine (Figure 1; see supplementary Table S1 for more details). The evacuated ^14^C- tryptophan that was recovered in the incubation seawater varied from 57% to 67% for the fish that were fed the 40P18L and 44P21L diets, respectively. The proportion of ^14^C-lysine that was evacuated ranged from 21% in fish that were fed the 44P21L diet to 33% in 44P18L fed fish. Concerning ^14^C-methionine evacuation, the proportion that was not absorbed varied from 27% to 42% in the fish that were fed the 44P18L and 44P21L diets, respectively. No significant differences were detected between lysine and methionine evacuation in fish fed all the experimental diets. The assessment of ^14^C-amino acid utilisation in gilthead seabream juveniles that were fed experimental diets with distinctive dietary protein and/or lipid content showed that the dietary treatments (44P21L, 44P18L, 40P21L, and 40P18L) had no influence on the evacuation of lysine, tryptophan, and methionine, but rather strongly dependent on their ketogenic and/or glucogenic nature.

**Figure 2:**
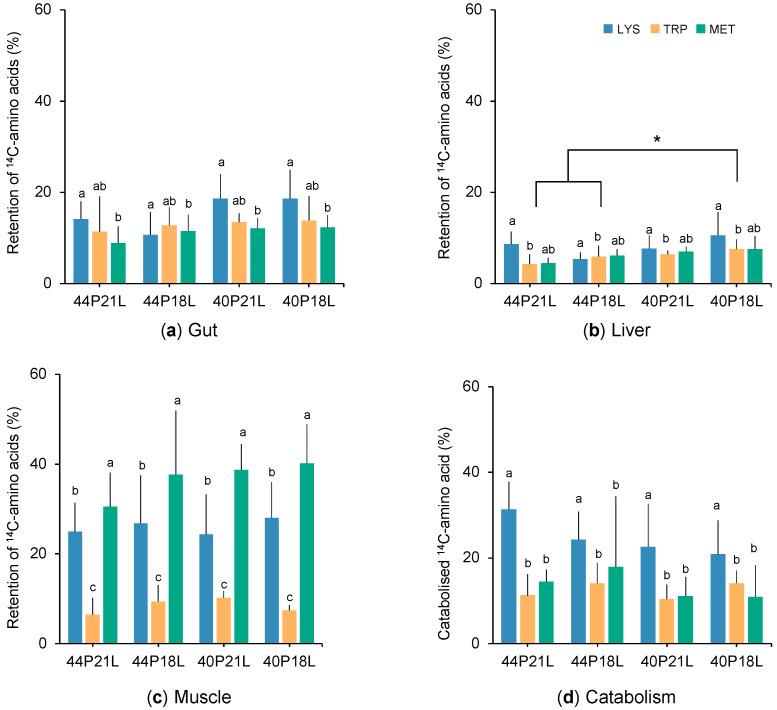
Proportion (%) of the total recovered ^14^C-amino acids lysine (LYS), tryptophan (TRP) and methionine (MET) that was retained in the: gut (**a**); liver (**b**); and muscle (**c**); or catabolised (**d**) in gilthead seabream juveniles fed 44P21L, 44P18L, 40P21L or 40P18L diets. Values are presented as mean ± standard deviation (*n* = 6 fish for each diet and tracer). Letters (a, b, c) represent significant differences among amino acids metabolic fate as a function of their nature, and * denote significant differences among dietary treatments (two-way ANOVA followed by Tukey’s multiple-comparison test, *p* < 0.05).

The amino acid retention in the gut (Figure 2a) was affected by the diet formulations, although post-hoc tests did not determine which treatments were significantly different. In general, fish that were fed the 40P21L and the 40P18L diets presented higher retention values for all amino acids than the fish that were fed the high protein diets. Additionally, the nature of the amino acid influenced their retention in this tissue. Lysine was significantly more retained than methionine in all dietary treatments. Tryptophan retention in the gut was similar to lysine or methionine.

The retention of the amino acids in the liver was influenced both by the amino acid nature and by the diet formulation (Figure 2b). Fish that were fed a higher amount of dietary protein (44P21L and 44P18L) presented lower amino acid retention in the liver, but only significantly different when compared to the diet with less protein and lipids (40P18L). Concerning the nature of the amino acids, lysine was significantly more retained in the liver than tryptophan, while methionine presented intermediate levels.

Methionine was preferentially retained in the muscle (Figure 2c). Although no significant differences were found among fish fed the different diets, the proportion of ^14^C-methionine retained in the muscle ranged from 30% in fish fed the 44P21L to 40% in 40P18L fed fish. Moreover, the retention of methionine in the muscle was significantly higher than lysine and tryptophan that presented mean retention values of 26% and 8%, respectively, for all dietary treatments.

The amino acids’ nature significantly influenced their catabolism, independently of the diet. Lysine was significantly more catabolised than tryptophan and methionine (Figure 2d). Catabolised lysine varied from approximately 21% in fish that were fed the 40P18L diet to 31% in 44P21L fed fish. The percentage of tube-fed ^14^C-tryptophan and ^14^C-methionine that was catabolised was around 12% and 14%, respectively, for all dietary treatments.

**Figure 3:**
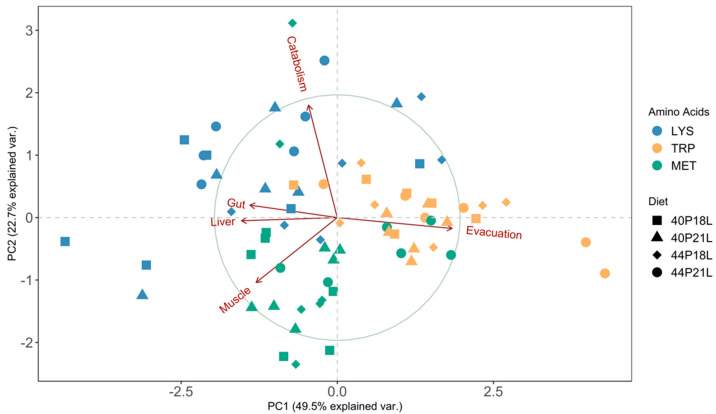
Principal component analysis (PCA) of the metabolic flux assays data generated by gilthead seabream juveniles fed different diets (44P21L, 44P18L, 40P21L, and 40P18L) labelled with ^14^C-amino acids lysine (LYS), tryptophan (TRP), and methionine (MET). Each point represents the projection of an individual sample in the PC1 and PC2 axis. Each dietary treatment is identified by a unique shape and each ^14^C-amino acid as a unique colour, as indicated in the legend.

A principal component analysis (PCA) was used to reduce the complexity of the data from the metabolic flux assays. The PCA confirmed that the nature of the amino acids, not the diet formulations, was responsible for the observed differences (Figure 3). PC1 and PC2 accounted for 49.5% and 22.7% of the total variability of the data. The analysis of the score plots indicated that the tryptophan data were separated from lysine and methionine along the PC1 axis, and that lysine and methionine were separated from each other along the PC2 axis. Evacuation and retention in the liver were the loadings that contributed the most for the dissimilarities observed between fish that were fed ^14^C-tryptophan labelled diets and fish that were fed diets labelled either with ^14^C-lysine or ^14^C-methionine. On the other hand, catabolism and muscle retention were the loadings responsible for the differences between fish that were fed ^14^C-lysine labelled diets and fish that were fed diets labelled with ^14^C-methionine.

**Figure 4:**
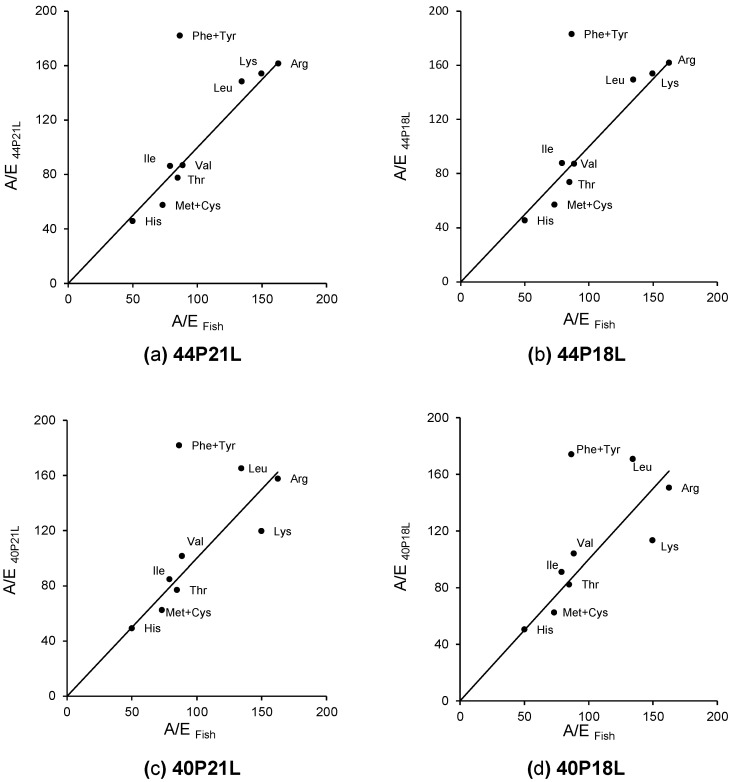
Comparison of the A/E ratios of the experimental diets: (**a**) 44P21L; (**b**) 44P18L; (**c**) 40P21L; (**d**) 40P18L with the A/E ratios of gilthead seabream juveniles.

Although the diets were supplemented with crystalline indispensable amino acids, a comparison of the dietary A/E ratios with the A/E ratios of fish revealed that all diets presented some amino acid imbalances (Figure 4). Lysine was balanced in the high protein diets 44P21L and 44P18L (Figure 4a,b); however, the lower protein diets, 40P21L and 40P18L (Figure 4c,d), presented a deficiency of 20% and 24%, respectively. Phenylalanine and tyrosine were found to be in excess in all diets (102% to 112%), while methionine and cysteine were deficient (~20% in the case of the higher protein diets and 14% for diets with lower protein content).

## 4. Discussion

Metabolic flux assays for ^14^C-lysine, ^14^C-tryptophan, or ^14^C-methionine were used to assess the bioavailability and metabolic fate of the selected indispensable amino acids as a function of their nature (ketogenic and/or glucogenic) and of the different dietary formulations in gilthead seabream juveniles. The metabolic budget of the selected amino acids was mainly affected by their nature rather than the diet formulation.

A principal component analysis (PCA) confirmed that the data were clustered by amino acid and not by dietary treatment. Additionally, the evacuation was the variable that contributed the most for the formation of two groups along the PC1 axis, one clustering observations from fish fed all diets labelled with ^14^C-tryptophan and another that clustered data from fish fed ^14^C-lysine and ^14^C-methionine labelled diets. In the current study, tryptophan evacuation was substantially higher than the evacuation of lysine and methionine (mean values of 60% versus 26% and 32%, respectively). Similarly, the metabolic budget determination in Senegalese sole (*Solea senegalensis*) juveniles and in white seabream (*Diplodus sargus*) larvae also revealed that a higher proportion of ^14^C-tryptophan was evacuated (41% and 30%, respectively) when compared to all the other indispensable amino acids [19] or to methionine and arginine [28]. The data on tryptophan digestibility is limited, yet juvenile rainbow trout (*Oncorhynchus mykiss*) showed a lower digestibility of tryptophan compared to all the other indispensable amino acids independently of the dietary treatment [29]. Free amino acids are transported across the brush-border membrane through carrier-mediated transport systems [4,30]. The same transporter may be responsible for the uptake of several amino acids and consequently some amino acids may interfere with the intestinal uptake of others [9,31]. TAT1 (Slc16a10) is responsible for the transport of the aromatic amino acids (phenylalanine, tyrosine, and tryptophan) in animal cells [32,33]. The calculation of the A/E ratios showed that in the present study all the experimental diets presented a phenylalanine and tyrosine content above the fish requirements. Therefore, it is reasonable to assume that a dietary excess of the two amino acids may interfere with the uptake of tryptophan since they all compete for the same transporter. The high levels of phenylalanine and tyrosine present in all the experimental diets might have potentially increased the evacuation of tryptophan resulting in its low availability for metabolic purposes. The current findings highlight the importance of feeding fish with balanced amino acid diets since not only deficiencies but also surpluses may have implications for amino acid absorption and utilisation.

The proportion of amino acids retained in the gut and liver of the seabream juveniles was affected not only by their nature but also by their dietary treatments. Fish that were fed the 40P18L diet retained more amino acids in the liver than fish that were fed both high protein diets (44P21L and 44P18L). These results suggest that when fish are fed the lower protein diets (40P21L and 40P18L), amino acids may be temporarily retained in the liver to ensure its availability for metabolic purposes. Furthermore, 18 h after feeding, lysine, which is considered an indicator of protein synthesis, was one of the most retained amino acids in the gut and liver of seabream juveniles. The relatively high retention in these tissues may be associated with the constant cellular turnover occurring in the former and with the crucial role of the latter in energy homeostasis [1]. In general, the tryptophan retention in the tissues was low: 13%, 6%, and 8% was found in the gut, liver, and muscle of juvenile seabream, respectively. It is worth mentioning that most of the retained tryptophan (±48%) was found in the gut, which was also observed in Senegalese sole juveniles [19]. This may be due to tryptophan being the precursor of 5-hydroxytryptophan, which in turn is the precursor of serotonin, a hormone mainly synthesised in the gastrointestinal tract [11,34], or due to the beneficial effects of tryptophan in the intestinal structural integrity of fish [35].

The present study showed that most of the absorbed methionine was retained in the muscle. This agrees with the results obtained with tracer studies in Senegalese sole juveniles and larvae, as well as in white seabream larvae [19,28,36,37]. Moreover, the current work revealed that the retention of methionine in the muscle was significantly higher than lysine and tryptophan retention. The PCA results corroborate these findings, demonstrating that fish that were fed all diets labelled with ^14^C-methionine were clustered together according to the data from the muscle retention. Dietary methionine supplementation was shown to improve growth performance in several fish species [16,38,39] and its involvement in muscle growth and in the regulation of the expression of genes related to myogenesis has been established [40]. Additionally, as a precursor of S-adenosylmethionine (SAM) and of polyamines, methionine is involved in DNA and protein methylation as well as in cell proliferation and differentiation [41,42]. The fact that methionine was preferentially retained in the muscle of fish that were fed all experimental diets reinforces the importance of methionine availability in this tissue for growth and other metabolic purposes. A recent study in seabream juveniles that were fed diets differing in the protein to energy ratios revealed that the higher retention of methionine in the muscle, when compared with protein or lysine, was due to its presence in the amino acid free pool and not in the protein-bound fraction, reinforcing the crucial role of methionine in various metabolic functions in addition to protein synthesis [18].

Inevitably, a fraction of the absorbed amino acids is catabolised and used for energy rather than growth [8,15]. This is especially true if imbalances in the dietary amino acid profile occur. Amino acid catabolism occurs mainly in the liver where amino acids are either converted into others or directed to the tricarboxylic acid (TCA) cycle. Once they enter the TCA cycle, amino acids can either be oxidised to generate energy, or channelled towards fatty acid synthesis or gluconeogenesis [9]. Although gluconeogenesis occurs in the liver, in fish, this pathway is thought to be less significant for amino acids compared to their oxidation [8,15,43]. Previous metabolic studies in gilthead seabream [44,45] and Senegalese sole [46] using a ^14^C-amino acid mixture demonstrated that only a small percentage of absorbed amino acids were converted into lipids or other metabolites, where glucose was included. Accordingly, the current findings revealed that 25% of the ^14^C-lysine, an amino acid that is solely ketogenic, was detected in the CO_2_-metabolic traps, significantly more than methionine (glucogenic) or tryptophan (ketogenic and glucogenic). Similarly, a metabolic budget determination in Senegalese sole juveniles revealed that lysine was significantly more catabolised than all the other indispensable amino acids [19]. According to the A/E ratio analysis, all experimental diets exhibited a deficit in sulphur amino acids (methionine + cysteine). Moreover, this analysis confirmed that the higher protein diets, 44P21L and 44P18L, met gilthead seabream’s requirements for lysine, and the low protein diets, 40P21L and 40P18L, were deficient in this amino acid. Nevertheless, lysine was significantly more catabolised than the other amino acids, independently of the diet. The PCA results confirmed that the data from the catabolism of the fish that were fed all the diets labelled with ^14^C-methionine and ^14^C-lysine grouped these amino acids in two different clusters along the PC2 axis. Fish have the ability to regulate amino acid metabolism with various enzymes that are responsible for adjusting the differential use of individual amino acids [43]. In fact, several studies have demonstrated that selective amino acid retention and catabolism occurs in fish [28,47,48,49]. The present results revealed that gilthead seabream juveniles discriminate between the use of different amino acids and that catabolism seems to be preferentially ketogenic.

## 5. Conclusions

In this study, the assessment of ^14^C-amino acid utilisation in gilthead seabream juveniles that were fed different diet formulations showed that amino acids’ bioavailability and metabolic fate were mainly determined by their nature. The higher tryptophan evacuation may be related to its affinity to intestinal transporters and interaction among different amino acids. Therefore, the low availability of tryptophan should be considered when optimising fish diet formulations. This in vivo approach allowed for the unravelling of interesting particularities in tryptophan metabolism, which are usually difficult to untangle in nutritional studies, mainly due to technical difficulties in quantifying tryptophan content. Methionine was found to be preferentially retained in the muscle, most likely to be used for growth. Catabolism was mainly ketogenic, independently of the diet and of any dietary amino acid imbalances. The in vivo nutrient flux approach is a valuable tool that allows for the fine-tuning of diet formulations, since the optimisation of diets considering the amino acids’ bioavailability will maximise protein retention in fish.

## Figures and Tables

**Table 1:** Formulation and proximate composition of experimental diets.

*Ingredients (%)*	44P21L	44P18L	40P21L	40P18L
Fishmeal SP ^a^	22.00	22.00	16.00	16.00
Fishmeal ^b^	5.00	5.00	5.00	5.00
Soy protein concentrate ^c^	6.40	6.00	6.90	6.40
Wheat gluten ^d^	7.00	6.40	6.00	6.00
Corn gluten ^e^	11.00	11.00	10.00	10.00
Soybean meal ^f^	12.00	12.00	12.00	12.00
Rapeseed meal ^g^	5.00	5.00	5.00	5.00
Sunflower meal ^h^	4.00	5.00	5.00	5.00
Wheat meal ^i^	2.90	4.90	7.00	10.50
Whole peas ^j^	3.00	4.00	5.00	5.00
Fish oil ^k^	10.60	8.52	10.85	9.00
Rapeseed oil ^l^	6.60	5.68	6.85	5.70
Vitamin and Mineral Premix ^m^	1.00	1.00	1.00	1.00
Vitamin E ^n^	0.10	0.10	0.10	0.10
Choline chloride ^o^	0.10	0.10	0.10	0.10
Betaine HCl ^p^	0.50	0.50	0.50	0.50
Soy lecithin ^q^	0.50	0.50	0.50	0.50
Guar gum ^r^	0.50	0.50	0.50	0.50
Antioxidant powder ^s^	0.20	0.20	0.20	0.20
Mono-calcium phosphate ^t^	1.10	1.10	1.40	1.40
L-Lysine ^u^	0.30	0.30	0.10	0.10
L-Threonine ^v^	0.20	0.20	-	-
*Proximate composition (% as fed)*				
Dry matter	94.17	93.73	94.69	94.05
Ash	8.53	9.22	8.10	8.20
Crude protein	44.72	43.88	40.40	40.43
Crude lipids	20.32	17.65	21.38	18.40
Total phosphorus	1.15	1.08	1.20	1.19
Gross energy (MJ kg^−1^)	22.33	21.71	22.11	21.66

All values are reported as means of duplicate analysis. ^a^ Super-Prime: 68% crude protein (CP), 8% crude fat (CF); Pesquera Diamante, Peru. ^b^ CONRESA 60: 65% CP, 10% CF; Conserveros Reunidos S.A., Spain. ^c^ Soycomil P: 63% CP, 8% CF; ADM, The Netherlands. ^d^ VITAL: 80% CP, 7.5% CF; Roquette Frères, France. ^e^ Corn gluten meal: 61% CP, 6% CF; COPAM, Portugal. ^f^ Solvent extracted dehulled soybean meal: 47% CP, 2.6% CF; CARGILL, Spain. ^g^ Defatted rapeseed meal: 34% CP, 2% CF; Premix Lda., Portugal. ^h^ Solvent extracted dehulled sunflower meal: 43% CP, 3% CF; MAZZOLENI SPA, Italy. ^i^ Wheat meal: 10% CP, 1.2% CF; Casa Lanchinha, Portugal. ^j^ Yellow peas: 19.6% CP, 2.2% CF; Ribeiro e Sousa Lda., Portugal. ^k^ Sopropêche, France. ^l^ J.C. Coimbra Lda., Portugal. ^m^ PREMIX Lda., Portugal: Vitamins (IU or mg kg^−1^ diet): DL-alpha tocoferol acetate 100 mg; sodium menadione bisulphate 25 mg; retinyl acetate 20,000 IU; DL-cholecalciferol 2000 IU; thiamin 30 mg; riboflavin 30 mg; pyridoxine 20 mg; cyanocobalamine 0.1 mg; nicotinic acid 200 mg; folic acid 15 mg; ascorbic acid 1000 mg; inositol 500 mg; biotin 3 mg; calcium panthotenate 100 mg; choline chloride 1000 mg; and betaine 500 mg. Minerals (g or mg k^−1^g diet): cobalt carbonate 0.65 mg; copper sulphate 9 mg; ferric sulphate 6 mg; potassium iodide 0.5 mg; manganese oxide 9.6 mg; sodium selenite 0.01 mg; zinc sulphate 7.5 mg; sodium chloride 400 mg; calcium carbonate 1.86 g; and excipient wheat middlings. ^n^ ROVIMIX E50, DSM Nutritional Products, Switzerland. ^o^ ORFFA, The Netherlands. ^p^ Beta-Key 95%, ORFFA, The Netherlands. ^q^ Lecico P700IPM, LECICO GmbH, Germany. ^r^ Guar gum, Seah International, France. ^s^ Paramega PX, KEMIN EUROPE NV, Belgium. ^t^ MCP: 22% P, 18% Ca; Fosfitalia, Italy. ^u^ L-Lysine HCl 99%; Ajinomoto Eurolysine SAS, France. ^v^ L-Threonine: 98%; EVONIK Nutrition & Care GmbH, Germany.

**Table 2:** Amino acid composition of experimental diets.

Amino Acids *(mg AA g^−1^ as fed)*	44P21L	44P18L	40P21L	40P18L
Arginine	45.1	42.9	31.4	29.8
Histidine	12.8	12.1	9.8	10.0
Lysine	43.0	40.8	23.8	22.4
Threonine	21.7	19.6	15.3	16.3
Isoleucine	24.1	23.3	16.9	18.0
Leucine	41.4	39.6	32.9	33.8
Valine	24.2	23.2	20.2	20.6
Tryptophan	n.d.	n.d.	n.d.	n.d.
Methionine	13.0	12.2	10.0	10.1
Phenylalanine	27.7	26.7	20.2	19.9
Cystine	3.1	3.0	2.5	2.3
Tyrosine	23.1	21.9	15.9	14.5
Aspartic acid + Asparagine	54.6	51.5	33.1	31.3
Glutamic acid + Glutamine	104.5	99.4	65.4	64.3
Alanine	26.3	24.9	21.0	20.6
Glycine	29.0	27.6	24.1	24.2
Proline	32.2	30.4	26.1	26.2
Serine	26.4	25.1	19.7	20.1

All values are reported as means of duplicate analysis. n.d.: not determined.

## Data Availability

The data presented in this study are available on request from the corresponding author.

## Supplementary Materials

The following supporting information can be downloaded at: https://www.mdpi.com/article/10.3390/ani12131713/s1, Table S1: Proportion (%) of the total recovered ^14^C-lysine (Lys), ^14^C-tryptophan (Trp) and ^14^C-methionine (Met) that was evacuated, retained in the gut, liver, and muscle, or catabolised in gilthead seabream juveniles fed 44P21L, 44P18L, 40P21L, or 40P18L diets.
