# Supplementary material for: Metabolic Fate Is Defined by Amino Acid Nature in Gilthead Seabream Fed Different Diet Formulations
Source: Animals (Basel). 2022 Jul 2;12(13):1713. doi: 10.3390/ani12131713 (PMC9264960; doi:10.3390/ani12131713)
Supplement: Supplementary file 1 [file animals-12-01713-s001.zip › animals-1764803-supplementary.pdf]

Table S1. Proportion (%) of the total recovered ^{14}C-lysine (Lys), ^{14}C-tryptophan (Trp) and ^{14}C-methionine (Met) that was evacuated, retained in the gut, liver and muscle, or catabolised in gilthead seabream juveniles fed 44P21L, 44P18L, 40P21L or 40P18L diets.

	**Dietary Treatment**														
**%**	**44P21L**			**44P18L**			**40P21L**			**40P18L**			**Two-way ANOVA (*P* < 0.05)**		
	LYS	TRP	MET	LYS	TRP	MET	LYS	TRP	MET	LYS	TRP	MET	AA	Diet	AA × Diet
**Evacuation**	20.9 ± 6.7 ^{b}	66.7 ± 17.5 ^{a}	41.6 ± 10.9 ^{b}	32.9 ± 11.9 ^{b}	58.0 ± 10.8 ^{a}	26.9 ± 6.0 ^{b}	26.8 ± 9.0 ^{b}	59.6 ± 3.2 ^{a}	31.3 ± 5.0 ^{b}	22.0 ± 15.5 ^{b}	57.2 ± 8.6 ^{a}	29.1 ± 6.5 ^{b}	***	NS	NS
**Gut**	14.1 ± 3.9 ^{a}	11.3 ± 7.7 ^{ab}	8.9 ± 3.6 ^{b}	10.7 ± 5.0 ^{a}	12.7 ± 4.0 ^{ab}	11.5 ± 3.6 ^{b}	18.6 ± 5.4 ^{a}	13.5 ± 1.9 ^{ab}	12.0 ± 2.3 ^{b}	18.6 ± 6.4 ^{a}	13.7 ± 5.4 ^{ab}	12.3 ± 2.7 ^{b}	*	*	NS
**Liver**	8.7 ± 2.7 ^{a,y}	4.3 ± 2.1 ^{b,y}	4.5 ± 1.0 ^{ab,y}	5.4 ± 1.5 ^{a,y}	5.9 ± 2.4 ^{b,y}	6.1 ± 1.4 ^{ab,y}	7.6 ± 2.9 ^{a,xy}	6.4 ± 0.8 ^{b,xy}	6.9 ± 1.1 ^{ab,xy}	10.6 ± 5.1 ^{a,y}	7.6 ± 2.1 ^{b,y}	7.6 ± 2.9 ^{ab,y}	*	**	NS
**Muscle**	24.9 ± 6.4 ^{b}	6.5 ± 3.7 ^{c}	30.5 ± 7.6 ^{a}	26.8 ± 10.7 ^{b}	9.4 ± 3.6 ^{c}	37.6 ± 14.3 ^{a}	24.4 ± 8.9 ^{b}	10.2 ± 1.5 ^{c}	38.6 ± 5.8 ^{a}	28.0 ± 8.0 ^{b}	7.4 ± 1.2 ^{c}	40.1 ± 8.7 ^{a}	***	NS	NS
**Catabolism**	31.3 ± 6.4 ^{a}	11.3 ± 5.0 ^{b}	14.5 ± 2.8 ^{b}	24.3 ± 6.5 ^{a}	14.0 ± 4.8 ^{b}	17.9 ± 16.4 ^{b}	22.6 ± 10.0 ^{a}	10.3 ± 3.4 ^{b}	11.1 ± 4.5 ^{b}	20.8 ± 7.9 ^{a}	14.1 ± 2.9 ^{b}	10.9 ± 7.4 ^{b}	***	NS	NS

Values are presented as mean ± standard deviation (n = 6 fish for each diet and amino acid). Within a row, superscript letters *a,b,c* represent significant differences in the metabolic fate of the distinct amino acids at each dietary treatment; *x,y* denote significant differences between dietary treatments (*P < 0.05; **P < 0.01; ***P < 0.001); NS: non-significant (P > 0.05).
